# *Delirium* and quality of life in critically ill patients: a prospective cohort study

**DOI:** 10.5935/0103-507X.20200072

**Published:** 2020

**Authors:** Lúcia Fabiane da Silva Luz, Moreno Calcagnotto dos Santos, Tiago Almeida Ramos, Clarissa Balbão de Almeida, Márcia Cristina Rover, Claudia Pellizzer Dal’Pizzol, Cristiane Letícia da Silva Pohren, Aline Vanessa da Silva Martins, Márcio Manozzo Boniatti

**Affiliations:** 1 Faculdades Integradas de Taquara - Taquara (RS), Brazil.; 2 Intensive Care Unit, Hospital de Clínicas de Porto Alegre - Porto Alegre (RS), Brazil.; 3 Department of Intensive Care Medicine, Hospital de Montenegro - Montenegro (RS), Brazil.; 4 Universidade La Salle - Canoas (RS), Brazil.

**Keywords:** Delirium, Quality of life, Patient discharge, Physical functional performance, Cognition, Intensive care units, Delirium, Qualidade de vida, Alta do paciente, Desempenho físico funcional, Cognição, Unidades de terapia intensiva

## Abstract

**Objective:**

To evaluate the association between the incidence of *delirium* in the intensive care unit and quality of life 1 month after hospital discharge.

**Methods:**

This was a prospective cohort study conducted in the intensive care units of two medium-complexity hospitals from December 2015 to December 2016. *Delirium* was identified using the Confusion Assessment Method for the Intensive Care Unit scale. At the time of hospital discharge, functional capacity and cognition were assessed with the Barthel index and the Mini Mental State Examination, respectively. Thirty days after patient discharge, the World Health Organization Quality of Life-BREF questionnaire was administered by telephone.

**Results:**

A total of 216 patients were included. *Delirium* was identified in 127 (58.8%) of them. Patients with *delirium* exhibited greater functional dependence (median Barthel index 50.0 [21.2 - 70.0] versus 80.0 [60.0 - 95.0]; p < 0.001) and lower cognition (Mini Mental State Examination score 12.9 ± 7.5 versus 20.7 ± 9.8; p < 0.001) at hospital discharge. There was no difference in any of the quality-of-life domains evaluated 1 month after hospital discharge between patients with and without *delirium*.

**Conclusion:**

Our findings suggest that patients with *delirium* in the intensive care unit do not have worse quality of life 1 month after hospital discharge, despite presenting greater cognitive impairment and functional disability at the time of hospital discharge.

## Abstract (pt)

**Objetivo:**

Avaliar a associação entre a incidência de *delirium* na unidade de terapia intensiva e qualidade de vida 1 mês após a alta hospitalar

**Métodos:**

Trata-se de estudo de coorte prospectivo desenvolvido em unidades de terapia intensiva de dois hospitais de média complexidade durante o período de dezembro de 2015 a dezembro de 2016. *Delirium* foi identificado por meio da escala *Confusion Assessment Method for the Intensive Care Unit*. No momento da alta hospitalar, foram avaliadas capacidade funcional e cognição por meio do índice de Barthel e da escala de Mini Exame do Estado Mental, respectivamente. Após 30 dias da alta hospitalar do paciente, por meio de contato telefônico, aplicou-se o questionário *World Health Organization Quality of Life-Bref*.

**Resultados:**

Foram incluídos 216 pacientes. *Delirium* foi identificado em 127 (58,8%) deles. Os pacientes com *delirium* apresentaram maior dependência funcional (mediana do índice de Barthel 50,0 [21,2 - 70,0] *versus* 80,0 [60,0 - 95,0]; p < 0,001) e menor cognição (escore do Mini Exame do Estado Mental 12,9 ± 7,5 *versus* 20,7 ± 9,8; p < 0,001) na alta hospitalar. Com relação à qualidade de vida, avaliada 1 mês após alta hospitalar, não houve diferença, em nenhum dos domínios, entre os pacientes com e sem *delirium*.

**Conclusão:**

Nossos achados sugerem que os pacientes com *delirium* na unidade de terapia intensiva não apresentam piora da qualidade de vida 1 mês após a alta hospitalar, apesar de apresentarem maior prejuízo cognitivo e incapacidade funcional no momento da alta hospitalar.

## INTRODUCTION

*Delirium* is an acute attentional disorder with cognitive changes and a fluctuating course, with or without hyperactive symptoms, that often occurs in critically ill patients. According to a meta-analysis of more than 16,000 critically ill patients, the incidence of *delirium* is almost one-third.^(1)^ Some studies, however, have reported rates higher than 80% in patients requiring mechanical ventilation (MV).^(2,3)^ In addition to longer hospital stay and higher mortality, patients with *delirium* have greater cognitive impairment and functional disability in the long term.^(4,5)^ Its association with quality of life, however, is still controversial. Two studies suggest that *delirium* is a risk factor for worse quality of life^(6,7)^ , while four other studies suggest that there is no association.^(8-11)^

The present study evaluated the association between the incidence of *delirium* in the intensive care unit (ICU) and quality of life 1 month after hospital discharge.

## METHODS

This was a prospective cohort study. The study was conducted in the ICUs of *Hospital de Aeronáutica de Canoas* (HACO), in Rio Grande do Sul, Brazil, a private medium-complexity hospital, and of the *Hospital de Montenegro*, also in Rio Grande do Sul, a public medium-complexity hospital. The HACO ICU had five beds for clinical and surgical patients separated by curtains, in a shared room, with windows that let in natural light. The ICU of *Hospital de Montenegro* had ten beds for clinical and surgical patients, two individual ones and eight separated by curtains in a shared room, with windows that let in natural light. In neither of the two ICUs was there a policy of extended family visitation during the data collection period. Visitation in both ICUs was allowed for 1 hour at three different times of day. The study was approved by the Research Ethics Committee of *Universidade La Salle*, located in Canoas, under CAAE no. 49738715.4.0000.5307.

All patients admitted to either ICU from December 2015 to December 2016 were assessed for eligibility. Patients to whom the Confusion Assessment Method for the Intensive Care Unit (CAM-ICU) scale could not be applied during the ICU stay or who stayed in the ICU for less than 24 hours were excluded.

The following variables were collected at admission to the ICU: age, sex, previous functional capacity according to the Barthel index (answered by a family member), origin of admission, type of admission and Simplified Acute Physiology Score 3 (SAPS 3). During the ICU stay, the need for MV, continuous sedation, use of vasopressors, and tracheostomy were recorded. *Delirium* was identified using the CAM-ICU, validated for Portuguese^(12)^ , which was applied twice a day throughout the patient’s stay in the ICU after assessing the patient’s level of sedation with the Richmond Agitation and Sedation Scale (RASS). The patient had to have a RASS score between -3 and +4 for the CAM-ICU to be applied. The Barthel index^(13)^ and the Mini Mental State Examination (MMSE)^(14)^ were applied at the time of hospital discharge. Thirty days after patient discharge, the World Health Organization Quality of Life-BREF (WHOQOL-BREF) questionnaire was administered by telephone.^(15)^

The functional capacity was assessed using the Barthel index. This index measures the level of independence in 10 self-care activities: feeding, bathing, grooming, dressing, bladder and bowel control, toilet use, walking on level surfaces, walking on stairs, and transfers from chair to bed and back. The score ranges from 0 to 100. Patients were considered dependent if they had a Barthel index < 60.^(16)^

Cognition was assessed by means of the MMSE. The score ranges from 0 to 30. Patients were classified as having normal cognition (score ^3^ 24), mild cognitive impairment (score between 19 and 23), or severe cognitive impairment (score between 0 and 18).^(17)^

The quality of life of patients was assessed using the WHOQOL-BREF questionnaire. It contains 26 questions divided into four domains: physical, psychological, social relationships, and environment.

### Statistical analysis

Continuous variables are expressed as the mean ± standard deviation (SD) or median and interquartile range (IQR). The categorical variables are expressed as absolute numbers and percentages. Student’s *t*-test or the Mann-Whitney test was used for continuous variables, and the chi-squared test was used for categorical variables. To adjust for potential confounders, covariates were selected a priori based on clinical plausibility for the occurrence of the outcome. These covariates included age, length of ICU stay, SAPS 3, Barthel Index at admission, and need for MV. These variables, in addition to the diagnosis of *delirium*, were included in the multiple linear regression models using forward selection. A separate multiple linear regression analysis was performed for each WHOQOL-BREF domain. The level of significance was set at 0.05. Statistical analysis was performed with the commercially available statistical program Statistical Package for Social Sciences (SPSS), version 22.0 (SPSS, Chicago, IL, USA).

## RESULTS

**Figure 1:**
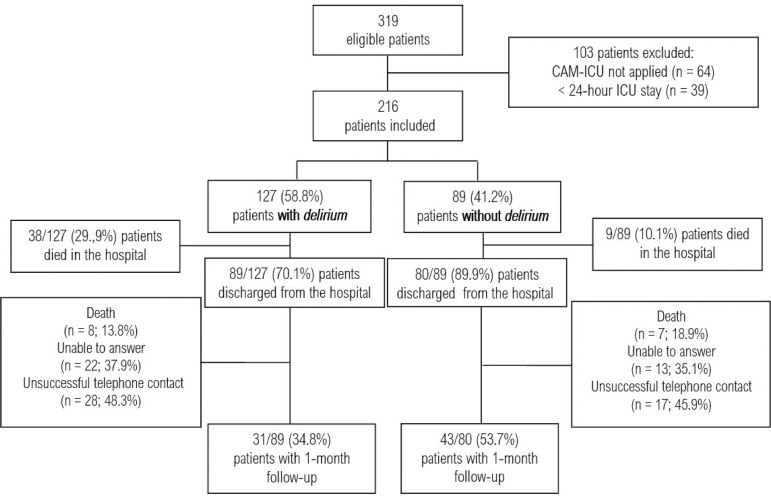
Flowchart of patient inclusion into the study. CAM-ICU - Confusion Assessment Method for the Intensive Care Unit; ICU - intensive care unit.

During the study period, 319 patients were evaluated for eligibility. A total of 103 patients were excluded, resulting in 216 patients for the study. *Delirium* was identified in 127 (58.8%) patients. Of the patients included in the study, 169 were discharged. Of these, 74 patients answered the quality of life questionnaire 1 month after hospital discharge (Figure 1).

Table 1 shows a descriptive analysis of patient characteristics. The patients with *delirium* were older and had a higher severity score and lower functional capacity before admission. In addition, during the ICU stay, these patients required MV more frequently and had longer ICU and hospital stays. Finally, patients with *delirium* had higher in-hospital mortality.

**Table 1:** Univariate comparison of the general patient characteristics according to the incidence of delirium during the intensive care unit stay

	With *delirium* (n = 127)	Without *delirium* (n = 89)	p value
Age (years)	67.4 ± 14.9	62.2 ± 15.7	0.014
Sex, male	65 (51.2)	55 (61.8)	0.122
Origin			0.021
Emergency	65 (51.2)	55 (61.8)	
Ward	29 (22.8)	20 (22.5)	
Surgical ward	6 (4.7)	8 (9.0)	
Another hospital	27 (21.3)	6 (6.7)	
Type of admission			0.125
Clinical	121 (95.3)	79 (88.8)	
Elective surgery	1 (0.8)	4 (4.5)	
Emergency surgery	5 (3.9)	6 (6.7)	
Barthel Index on admission	80.0 (60.0 - 100.0)	90.0 (70.0 - 100.0)	0.043
SAPS 3	66.4 ± 15.2	51.1 ± 15.5	< 0.001
MV	107 (84.3)	24 (27.0)	< 0.001
Tracheostomy	16 (12.6)	1 (1.1)	0.001
Continuous sedation	78 (61.4)	18 (20.2)	< 0.001
Benzodiazepine	33 (26.0)	3 (3.4)	< 0.001
Vasopressor	110 (86.6)	26 (29.2)	< 0.001
Length of ICU stay (days)	6.0 (4.0 - 10.0)	3.0 (2.0 - 4.0)	< 0.001
Length of hospital stay (days)	16.0 (9.0 - 26.0)	9.0 (6.0 - 14.0)	< 0.001
Death in the ICU	15 (11.8)	6 (6.7)	0.216
Death in the hospital	38 (29.9)	9 (10.1)	0.001

SAPS 3 - Simplified Acute Physiology Score 3; MV - mechanical ventilation; ICU - intensive care unit.

Regarding the duration of *delirium*, 49 (38.6%) patients had *delirium* for only 1 day, and 78 (61.4%) patients had *delirium* for more than 1 day.

Regarding functional capacity, in the univariate analysis, patients with *delirium* showed greater functional dependence than patients without *delirium*. The median Barthel index at hospital discharge was 50.0 (21.2 - 70.0) and 80.0 (60.0 - 95.0) for patients with and without *delirium*, respectively (p < 0.001). Among the patients with *delirium*, 30 (57.7%) were considered dependent. Of the patients without *delirium*, only 14 (21.2%) were considered dependent. In addition, 44 (84.6%) patients with *delirium* showed a decrease in the Barthel Index during their hospital stay. Among the patients without *delirium*, 28 (42.4%) showed a decrease (p < 0.001). In the multiple linear regression model, *delirium* maintained an independent association with functional capacity (Table 2).

**Table 2:** Multiple linear regression for functional capacity

	β coefficient	Standard error	p value
Barthel Index on admission	0.77	0.07	< 0.001
*Delirium*	-13.34	3.88	0.001
MV	-12.29	3.85	0.002

MV - mechanical ventilation. Model adjusted for age, length of intensive care unit stay and Simplified Acute Physiology Score 3.

Patients with *delirium* also had worse cognition at hospital discharge than patients without *delirium*. In the univariate analysis, the mean MMSE score of patients with *delirium* was 12.9 ± 7.5; for patients without *delirium*, the mean score was 20.7 ± 9.8 (p < 0.001). Of the 53 patients with *delirium* evaluated by the MMSE at hospital discharge, only two (3.8%) patients had normal cognition, and 40 (75.5%) had severe cognitive impairment. Among the 65 patients without *delirium* evaluated by the MMSE, the prevalence of normal cognition and severe cognitive impairment was 41.5% (n = 27) and 36.9% (n = 24), respectively. *Delirium* maintained an independent association with cognition in multiple linear regression (Table 3).

**Table 3:** Multiple linear regression for cognition

	β coefficient	Standard error	p value
Barthel Index on admission	0.12	0.02	< 0.001
*Delirium*	-5.69	1.14	< 0.001
SAPS 3	-0.09	0.04	0.03

SAPS 3 - Simplified Acute Physiology Score 3. Model adjusted for age, mechanical ventilation and length of intensive care unit stay.

Regarding quality of life evaluated 1 month after hospital discharge, there was no difference in the univariate analysis in any of the domains between patients with and without *delirium* (Table 4). The presence of *delirium* in the ICU was not correlated with quality of life after adjustment for confounders in the multiple linear regression model (Supplementary material). The reasons for loss to follow-up 1 month after hospital discharge were death (with *delirium*: 7, 18.9%; without *delirium*: 8, 13.8%; p = 0.80), cognitive and/or functional inability to answer the questionnaire by telephone (13, 35.1%; 22, 37.9%; p = 0.80), and inability to contact the patient by telephone (17, 45.9%; 28, 48.3%; p = 0.63).

**Table 4:** Comparison of the domains of the World Health Organization Quality of Life-BREF questionnaire between patients with and without *delirium*

	With *delirium* (n = 31)	Without *delirium* (n = 43)	p value
Physical	46.8 ± 23.6	50.8 ± 23.7	0.47
Psychological	53.4 ± 24.1	62.7 ± 20.0	0.08
Social relationships	48.9 ± 15.8	56.4 ± 18.8	0.08
Environment	56.4 ± 13.5	62.1 ± 16.2	0.12

## DISCUSSION

In this prospective cohort study involving a general population of critically ill patients, we found *delirium* was associated with decreased functional capacity and cognition, even after adjusting for confounding variables. However, *delirium* was not associated with quality of life 1 month after hospital discharge.

Few studies have evaluated the impact of the incidence of *delirium* during ICU stay on the quality of life of survivors after hospital discharge. Two studies suggest that *delirium* is a risk factor for worse quality of life,^(6,7)^ while four other studies suggest that there is no association.^(8-11)^ Those studies do not include the study by Jackson et al.^(18)^ because these authors evaluated only two of the eight domains of the Medical Outcomes Short-Form Health Survey. Van Rompaey et al.^(6)^ did not correct for disease severity. In the study by Abelha et al.,^(7)^ only surgical patients were included, which makes it difficult to generalize the results. In three of the four studies that did not find an association between *delirium* and quality of life, most patients included were surgical patients.^(8-10)^ In addition, two of these studies were single-center studies.^(8,10)^ Our study reinforces the findings in clinical patients and was performed in two centers.

In previous studies, quality of life was assessed between the second and 18th months after ICU discharge.^(6-11)^ The difference between patients with and without *delirium* in the ICU is likely to be more pronounced at the beginning of the recovery process.^(8)^ We chose to perform an earlier assessment to investigate an aspect not yet addressed in previous studies and to test the hypothesis that the first month after hospital discharge is the period when the greatest impact of *delirium* in the ICU is experienced. However, even with this early assessment in the course of recovery, we found no association of *delirium* with any of the domains evaluated on the quality-of-life scale.

After ICU stay, many patients have decreased functional capacity, even though they are functionally independent before admission.^(19,20)^ This impairment is usually seen in the ability to perform basic activities of daily living, such as bathing, dressing, eating, and bowel and bladder control, and it might be even greater when the patient experiences *delirium* in the ICU. Brummel et al. found an independent association between *delirium* and decreased functional capacity 1 year after ICU discharge.^(4)^ Similar results were found in another study, which included only surgical patients.^(7)^ However, the study that evaluated this outcome with the largest number of included patients did not observe such an association.^(18)^ In our study, patients with *delirium* showed more impaired functional capacity at hospital discharge, even after adjusting for confounding variables, including functional capacity before admission. Again, these discrepancies could be related to population characteristics and follow-up time. Our earlier assessment over the course of recovery most likely influenced our findings.^(21)^

The mechanisms responsible for the possible relationship between *delirium* and functional disability are still unclear. A potential mechanism is the reduction in spontaneous physical activity as part of hypoactive *delirium*, the most common *delirium* subtype among critically ill patients.^(22,23)^ This reduction in spontaneous physical activity can lead to muscle atrophy due to disuse and, later, to functional disability in the months after the critical illness.^(4,24,25)^ Another suggested mechanism is that inflammation, usually present in critically ill patients with *delirium*, leads to muscle mass loss in these patients.^(26)^

The association between *delirium* and cognitive impairment is more consistently demonstrated. Several multicenter prospective studies^(5,8,10,17)^ and a meta-analysis^(1)^ confirmed this association. In our study, most patients with *delirium* had severe cognitive impairment at the time of hospital discharge. Although that time may be too early to perform cognition assessment, a previous study found that cognitive function at the time of hospital discharge was a significant predictor of long-term cognitive function.^(27)^

The mechanisms responsible for the association between *delirium* and cognitive impairment are also unclear. *Delirium* is associated with reduced white matter integrity in the central nervous system, which is associated with cognitive impairment.^(28)^ In addition, *delirium* has been associated with cerebral atrophy, possibly through neuronal inflammation and apoptosis.^(29,30)^

Interestingly, cognitive impairment had no impact on the perceived quality of life of this population. Although an association between cognitive problems and worsened quality of life was expected, our results are consistent with previous studies that did not find this association.^(8,10)^

Our study has some limitations. The small number of included patients, especially patients who completed the 1-month follow-up, is an important limitation. Although the reasons for loss to follow-up were not different between patients with and without *delirium*, the amount of loss to follow-up may have added a bias to the results. Most of the losses were due to lack of telephone contact, and the patients we could not contact may have had worse quality of life, with less social support, than those who were contacted. Another limitation was the loss of patients due to the inability to apply the CAM-ICU. Most of these patients remained sedated until death, which made it impossible to apply the tool. In addition, the assessment of functional capacity before hospitalization was based on the report of family members, although this is a limitation of almost all studies with critically ill patients. Quality of life was not assessed before hospitalization, which is another limitation of the study. Lastly, we did not evaluate the long-term impact of *delirium*. Despite being a limitation, the earlier evaluation provides some findings not sought by other studies.

## CONCLUSION

Our findings suggest that patients with *delirium* in the intensive care unit do not have worsened quality of life 1 month after hospital discharge, despite presenting greater cognitive impairment and functional disability at the time of hospital discharge.
