# Supplementary material for: Delirium and quality of life in critically ill patients: a prospective cohort study
Source: Rev Bras Ter Intensiva. 2020 Jul-Sep;32(3):426–32. doi: 10.5935/0103-507X.20200072 (PMC7595710; doi:10.5935/0103-507X.20200072)
Supplement: Supplementary file 1 [file rbti-32-03-0426-suppl01.pdf]

Delirium and quality of life in critically ill patients: a prospective cohort study

Delirium e qualidade de vida em pacientes críticos: um estudo de coorte prospectivo

Lúcia Fabiane da Silva Luz[1], Moreno Calcagnotto dos Santos[2], Tiago Almeida Ramos[2], Clarissa Balbão de Almeida[3], Márcia Cristina Rover[3], Claudia Pellizzer Dal'Pizzol[3], Cristiane Letícia da Silva Pohren[3], Aline Vanessa da Silva Martins[3], Márcio Manozzo Boniatti[4]

Table 1S - Multiple linear regression for the physical domain of the World Health Organization Quality of Life-BREF questionnaire

	β coefficient	Standard error	p value
Barthel Index on admission	0.32	0.14	0.03

Adjusted for *delirium*, Simplified Acute Physiology Score 3, age, mechanical ventilation and days of intensive care unit stay.

Table 2S - Multiple linear regression for the psychological domain of the World Health Organization Quality of Life-BREF questionnaire

	β coefficient	Standard error	p value
Barthel Index on admission	0.46	0.13	0.001
Age	0.71	0.21	0.001
SAPS 3	-0.42	0.19	0.03

SAPS 3 - Simplified Acute Physiology Score 3. Adjusted for *delirium*, mechanical ventilation and days in the intensive care unit.

Table 3S - Multiple linear regression for the social relationships domain of the World Health Organization Quality of Life-BREF questionnaire

	β coefficient	Standard error	p value
SAPS 3	-0.35	0.14	0.02

SAPS 3 - Simplified Acute Physiology Score 3. Adjusted for Barthel Index at admission, *delirium*, age, mechanical ventilation and days in the intensive care unit.

Table 4S - Multiple linear regression for the environment domain of the World Health Organization Quality of Life-BREF questionnaire

	β coefficient	Standard error	p value
Barthel on admission	0.29	0.1	0.005
Age	0.46	0.15	0.004

Adjusted for *delirium*, mechanical ventilation, Simplified Acute Physiology Score 3 and days in the intensive care unit.
